# Non-RVD mutations that enhance the dynamics of the TAL repeat array along the superhelical axis improve TALEN genome editing efficacy

**DOI:** 10.1038/srep37887

**Published:** 2016-11-24

**Authors:** Naoya Tochio, Kohei Umehara, Jun-ichi Uewaki, Holger Flechsig, Masaharu Kondo, Takehisa Dewa, Tetsushi Sakuma, Takashi Yamamoto, Takashi Saitoh, Yuichi Togashi, Shin-ichi Tate

**Affiliations:** 1 Research Center for the Mathematics on Chromatin Live Dynamics (RcMcD), Hiroshima University, 1-3-1 Kagamiyama, Higashi-Hiroshima 739-8526, Japan; 2 Department of Mathematical and Life Sciences, Graduate School of Science, Hiroshima University, 1-3-1 Kagamiyama, Higashi-Hiroshima 739-8526, Japan; 3 Department of Life Science and Applied Chemistry, Nagoya Institute of Technology, Gokiso-cho, Showa-ku, Nagoya 466-8555, Japan; 4 Faculty of Pharmaceutical Sciences and Center for Research and Education on Drug Discovery, Hokkaido University, Kita-ku, Sapporo 060-0812, Japan

## Abstract

Transcription activator-like effector (TALE) nuclease (TALEN) is widely used as a tool in genome editing. The DNA binding part of TALEN consists of a tandem array of TAL-repeats that form a right-handed superhelix. Each TAL-repeat recognises a specific base by the repeat variable diresidue (RVD) at positions 12 and 13. TALEN comprising the TAL-repeats with periodic mutations to residues at positions 4 and 32 (non-RVD sites) in each repeat (VT-TALE) exhibits increased efficacy in genome editing compared with a counterpart without the mutations (CT-TALE). The molecular basis for the elevated efficacy is unknown. In this report, comparison of the physicochemical properties between CT- and VT-TALEs revealed that VT-TALE has a larger amplitude motion along the superhelical axis (superhelical motion) compared with CT-TALE. The greater superhelical motion in VT-TALE enabled more TAL-repeats to engage in the target sequence recognition compared with CT-TALE. The extended sequence recognition by the TAL-repeats improves site specificity with limiting the spatial distribution of FokI domains to facilitate their dimerization at the desired site. Molecular dynamics simulations revealed that the non-RVD mutations alter inter-repeat hydrogen bonding to amplify the superhelical motion of VT-TALE. The TALEN activity is associated with the inter-repeat hydrogen bonding among the TAL repeats.

Transcription activator-like effector (TALE) proteins secreted by plant bacterial pathogens bind to specific host promoters^1^,^2^,^3^,^4^,^5^. The DNA binding region of the TALE protein constitutes a unique array of repeats (TAL-repeat). These 34-residue repeats share a highly conserved sequence, except for two central residues at positions 12 and 13, which are denoted as the repeat variable diresidue, RVD^2^,^6^,^7^,^8^ (Fig. 1a and b). In binding to DNA, an array of TALE RVDs ensure sequence specificity^2^,^6^,^7^,^8^. The code relating RVDs to target DNA bases is established^6^,^7^. This modularity is exploited to generate TALE proteins tailored to recognise prescribed DNA sequences of interest^6^,^7^. An artificial fusion protein composed of a custom TAL-repeat array and the sequence independent FokI nuclease domain that functions as a dimer^9^ is called TALEN, and this protein is used widely as a genome editing tool^10^,^11^.

The DNA binding ability of each TAL-repeat in the array is position-dependent. Atypical N-terminal repeats prior to the canonical TAL-repeat array in TALE are essential for binding to DNA^12^. The atypical repeats bind to DNA in a sequence-independent manner and function as a fastening site to initiate formation of the TALE-DNA complex (Fig. 1c)^12^. The sequence-specific binding ability of each canonical TAL-repeat decreases along the position of the array: the repeats located at the N-terminus exhibit increased affinity^13^.

The structural plasticity associated with the tandem repeat array architecture of TALE is remarkable. The crystal structures of dHax3 TALE in the DNA-free and the DNA-bound states demonstrated that TALE becomes compressed upon binding to DNA^14^; the axis along which the TALE structure changes is defined as the superhelical axis (Supplementary Fig. S1). The substantial structural compression upon DNA binding was also observed in a solution by small angle X-ray scattering^15^. With coarse-grained elastic network models, TALE was shown to have preferential deformability along the superhelical axis, as anticipated from its coil spring-like shape^16^. An all-atom molecular dynamics (MD) simulation with principal component analysis consistently demonstrated that TALE has an open–close motion that adjusts the superhelical assembly of the TAL-repeats to achieve the sequence-specific recognition^17^.

A TALEN with periodical mutations at positions 4 and 32 (non-RVD residues) in each TAL-repeat increases the genome editing efficacy^18^,^19^,^20^ (Fig. 1a). The non-RVD residues are at the inter-repeat sites opposite the base recognition RVD loop (Fig. 1b); therefore, the residues do not directly affect the DNA binding. The molecular basis for the raised efficacy of the TALEN with the non-RVD mutations remains elusive. Characterising the action of the non-RVD mutations should advance our understanding of the TALE-DNA interaction and facilitate the design of TALENs with increased efficacy.

In this study, we compared the physicochemical properties between TALEs with and without the periodical mutation at non-RVD positions, hereafter referred to as VT-TALE and CT-TALE, respectively (Fig. 1a). VT and CT stand for ‘Variable Two residues’ and ‘Constant Two residues’ at non-RVD positions, respectively (Fig. 1c). Dynamic light scattering (DLS) and size exclusion chromatography (SEC) demonstrated that VT-TALE adopts a wider range of conformation compared with that of CT-TALE. This finding indicates that VT-TALE has greater amplitude motions along the superhelical axis (superhelical motion) over CT-TALE. The thermodynamic parameters for CT- and VT-TALEs in binding to DNA demonstrated that a larger number of TAL-repeats in VT-TALE engage in the DNA binding compared with that for CT-TALE. The extended sequence recognition achieved by VT-TALE could explain the increased genome editing efficacy of the TALEN comprising VT-TALE as the DNA binding domain.

## Results

### CT-TALE adopts a more elongated shape than VT-TALE

**Figure 1:**
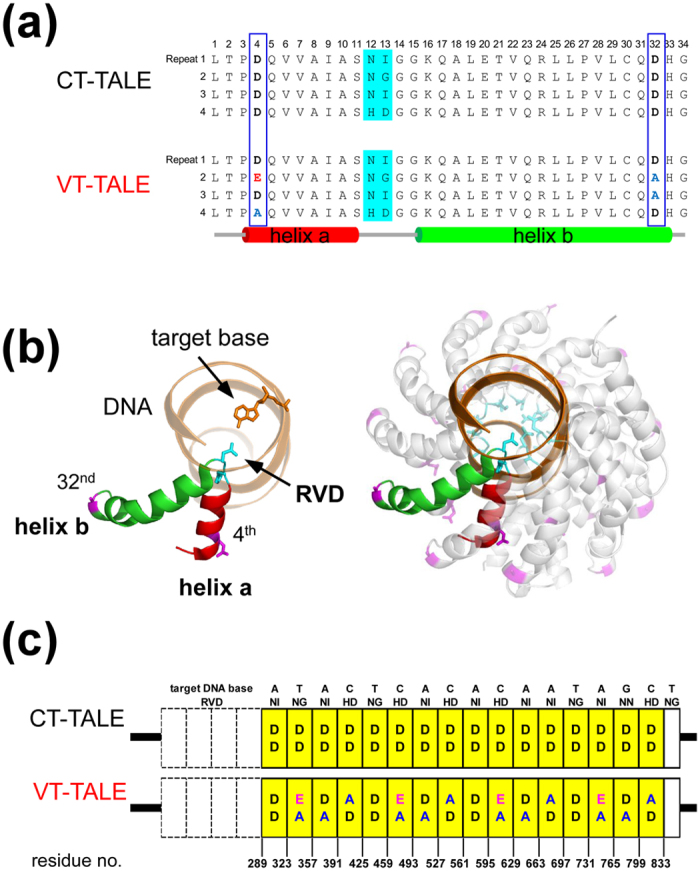
TALE sequence and structure. (**a**) Comparison between the amino acid sequences of the first 4 TAL-repeats of TALE with/without the periodically mutated repeats. The sequences of TAL-repeats without (CT-TALE, upper) and with the periodical mutations (VT-TALE, lower) are presented. RVDs are shaded in cyan. Non-RVD residues at the 4th and 32nd positions are presented as bold letters with different colours for the mutation sites. The secondary structures are presented at the bottom. (**b**) Ribbon representations of a single-TAL repeat (left) and the DNA bound TALE protein (right) (PDBID: 3V6T^14^). The first short helix (helix a) and the second long helix (helix b) are coloured red and green, respectively. RVD and the periodical mutation sites are noted in cyan and magenta, respectively, with the side chains shown as a stick representation. In the right panel, RVDs and the mutation sites are also displayed. DNA is also presented in orange. Figures were prepared using PyMOL (DeLano Scientific, San Carlos, CA). (**c**) Schematic representation of CT-TALE (upper) and VT-TALE (lower). TAL-repeats are represented as yellow boxes. Non-canonical pseudo-repeats^12^ and the last half repeats^30^ are presented as dashed boxes and white boxes, respectively. Amino acid types of non-RVD residues at the 4th and 32nd positions in each repeat are given as letters; mutated sites are coloured.

**Figure 2:**
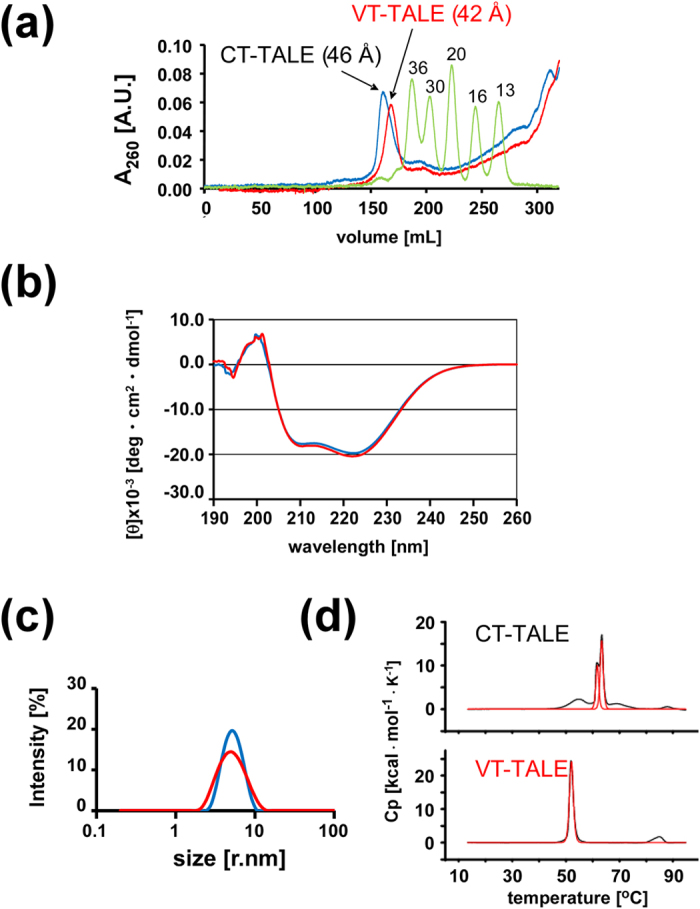
Comparison of physicochemical properties between CT-TALE and VT-TALE. (**a**) Analyses of size exclusion chromatograms of TALEs. Calibration of the column was performed using a standard globular protein set (GE Healthcare), and the chromatogram is presented in green. Stokes radii of the standard proteins are labelled (Å unit)^27^. The chromatograms of CT-TALE and VT-TALE are indicated in blue and red, respectively. Estimated Stokes radii of CT-TALE and VT-TALE are shown in parentheses. (**b**) CD spectra of TALEs. CD spectra of CT-TALE and VT-TALE are presented in blue and red, respectively. (**c**) Size distributions of CT- and VT-TALE. Particle size distributions of CT- and VT-TALE are presented in blue and red, respectively. Estimated Z-average sizes were 48.3 ± 0.7 Å and 44.7 ± 1.6 Å, respectively. (**d**) DSC thermograms of the CT- and VT-TALEs. Experimental data are shown in black, and fits for individual and composite fits are indicated in red. (upper) CT-TALE: *T*^1^_m_ = 61.71 ± 0.02 °C; *T*^2^_m_ = 63.41 ± 0.01 °C, (lower) VT-TALE: *T*_m_ = 52.07 ± 0.01 °C.

The CT-TALE and VT-TALE proteins used in this study have 16.5 TAL repeats and four atypical repeats at the N-terminus (Fig. 1c). Both TALEs share the same array of RVDs (Fig. 1c). SEC analysis showed that CT-TALE is larger in size than VT-TALE; the Stokes radii (*R*_S_) for CT-TALE and VT-TALE were estimated to be 46 Å and 42 Å, respectively (Fig. 2a). The radius of a globular protein with the same molecular weight, 80 kDa, is 28 Å^21^. The elucidated radii for CT- and VT-TALEs demonstrate that they adopt elongated shapes, as established by the crystal structures of TALEs^15^,^21^,^22^,^23^.

CT-TALE and VT-TALE demonstrated essentially the same CD spectra at 25 °C (Fig. 2b), implying that the non-RVD mutations did not change the secondary structures of the TAL-repeats and the superhelicity of the TAL-repeat array in VT-TALE (Fig. 2b). DLS experiments showed that the molecular size distribution of VT-TALE differed from CT-TALE (Fig. 2c): mean size = 48.3 ± 0.7 Å, polydispersity index (PDI) = 0.10 ± 0.03 (CT-TALE) and mean size = 44.7 ± 1.6 Å, PDI = 0.18 ± 0.02 (VT-TALE). The DLS results showed that both TALEs were monomodal but showed significant polydispersity with PDI values ≥0.1. Moreover, the average size of VT-TALE was smaller than CT-TALE (Fig. 2c), which is consistent with the SEC results (Fig. 2a). The force-probe simulation of the TAL-repeat array^16^ demonstrated the motion of the array preferentially variates the size of the longer semiaxis in its rod-like shape, as shown by the TALE crystal structures with and without DNA (Supplementary Fig. S1)^14^. The size distribution for the TALE under monodisperse conditions, therefore, is supposed to represent the variations in the TALE size along the superhelical axis. The greater polydispersity for VT-TALE suggests that the non-RVD mutations amplify the magnitude of the superhelical motion to generate a wider range of conformations.

Differential scanning calorimetry (DSC) experiments demonstrated that VT-TALE is structurally unstable relative to CT-TALE. The transition temperature (*T*_m_) of VT-TALE was 52.07 ± 0.01 °C, whereas CT-TALE exhibited two primal transition points at *T*^1^_m_ = 61.71 ± 0.02 °C and *T*^2^_m_ = 63.41 ± 0.01 °C, with broader peaks near 55 °C and 70 °C (Fig. 2d). The broader peaks suggest that the partially unfolded components occur during the thermal denaturation. The CD spectral change at 222 nm according to the raising temperature revealed that CT-TALE undergoes multiple structural states in the denaturing process, which include partially unfolded components (Supplementary Fig. S2). The large difference between the calorimetric and van’t Hoff enthalpies for the thermal denaturation of CT-TALE suggests that partially unfolded states are noted in its denaturing process (Supplementary Table S1). It was also the case for the VT-TALE thermal denaturing; significant discrepancy was observed between the calorimetric and van’t Hoff enthalpy values (Supplementary Table S1). Although the DSC data for VT-TALE exhibited single peaks, the thermal denaturing of VT-TALE is not a simple two-state structure transition (Fig. 2d).

### Changes in the inter-repeat interactions revealed by MD simulations

All-atom MD simulations were used to explore the changes in structure and dynamics of the TAL-repeat array caused by the non-RVD mutations. In the present simulation, we considered 11.5 TAL-repeats because CT- and VT-TALE structures were constructed from the dHax3 crystal structure comprising 11.5 TAL-repeats (Supplementary Fig. S3). The MD simulations revealed that CT- and VT-TALE became elongated to close to the extend forms in 50 ns, starting from the compressed DNA bound form modelled after the crystal structure of dHax3 in the complex with DNA^14^ (Supplementary Fig. S4).

**Figure 3:**
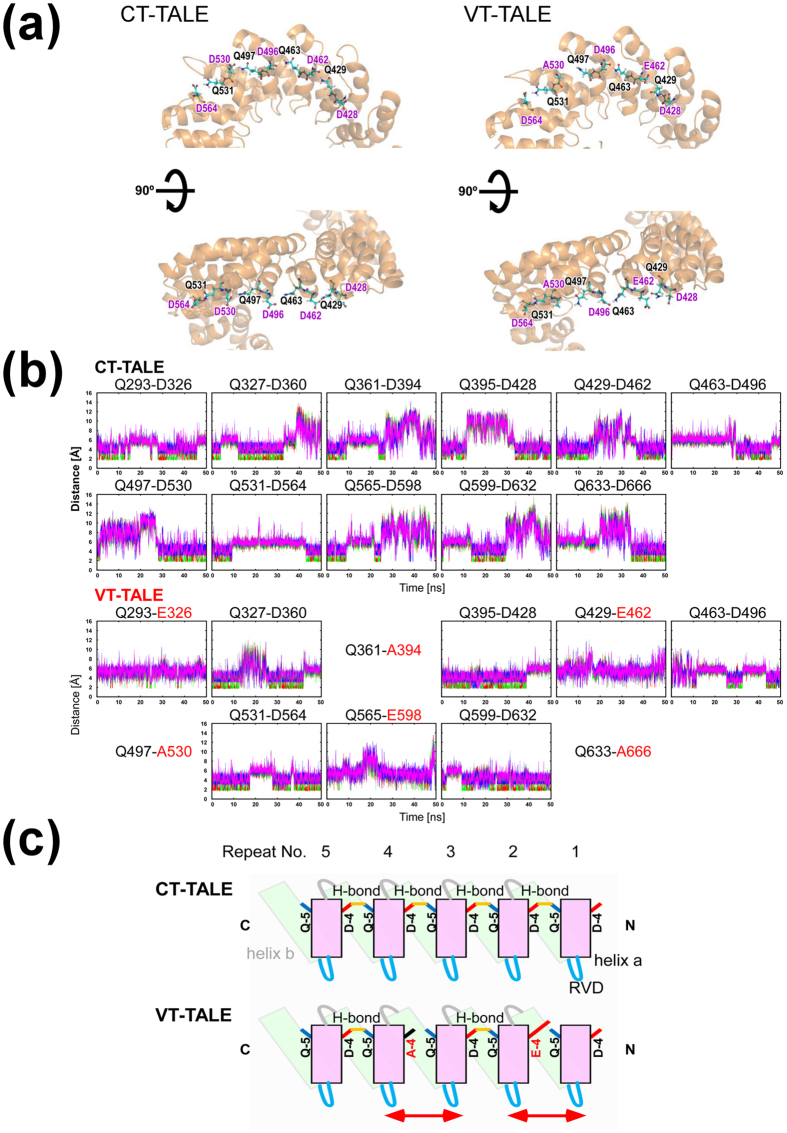
Inter-repeat hydrogen bonds among the TAL-repeats. (**a**) Snapshots of the CT- and VT-TALE models in the extended forms sampled at 50 ns in the MD trajectories. The structures are viewed from the C-terminal side of the superhelical structure and its 90-degree rotated position. For the four TAL-repeats in the centre of the array, the side chains of the residues positioned at 4 and 5 in each repeat of CT- and VT-TALEs are drawn as ball and stick representations. Residue at position 4, a non-RVD residue, in each TAL-repeat is marked in purple. For details of the modelling and simulation, see the Supplementary Information. (**b**) Distances between HE21/HE22 in Q-5 and OD1/OD2 in D-4 or OE1/OE2 in E-4 in the structures in the MD trajectory (corresponding to Trial 1 in Supplementary Fig. S4); every atomic pair is considered. (**c**) Schematic representation of the inter-repeat hydrogen bonds. Helices a and b and RVD are presented as magenta boxes, green boxes and cyan lines, respectively. The 4th and 5th amino acids in each TAL-repeat are represented by short lines. Hydrogen bonds are indicated by orange lines. In VT-TALE, two of the inter-repeat hydrogen bonds are not formed in a unit with four TAL-repeats.

Residues D-4 and Q-5 in the adjacent repeats were close to each other in the TAL-repeat array (Fig. 3a); the hyphenated residue name refers to the position within each TAL-repeat (Fig. 1a). As seen in the MD trajectories for CT-TALE, the hydrogen bonding donor and acceptor side chain atoms of D-4 and Q-5 stochastically become close to enable the inter-repeat hydrogen bond in the nanosecond time regime (Fig. 3b). The probability for the inter-repeat hydrogen bonding among the 11 sites in the extended CT-TALE (MD trajectory during 40–50 ns) was 12.1% (Supplementary Table S2).

In the extended VT-TALE (trajectory: 40–50 ns), the non-RVD mutations to D-4 reduced the inter-repeat hydrogen bonding probability. A-4 lacks the acceptor side chain oxygen to Q-5, whereas E-4 severely diminished the ability to form a hydrogen bond to Q-5 (Fig. 3b). The probability for the inter-repeat hydrogen bonding among all the pairs of D/E/A-4 and Q-5 in the extended VT-TALE was 7.1% (Supplementary Table S2).

The residues D-4 and Q-5 remained close to enable the inter-repeat hydrogen bonding in the compressed forms of CT- and VT-TALE (Supplementary Fig. S5). The inter-repeat hydrogen bonding probabilities for compressed CT- and VT-TALEs (trajectory: 0–10 ns) were 11.9% and 5.6%, respectively (Supplementary Table S2).

The TALEs used in this work include the units with each having four TAL-repeats (Fig. 1a). The inter-repeat hydrogen bonding sites in a unit of CT- and VT-TALE identified in the MD simulation are summarised schematically (Fig. 3c). CT-TALE has four potential sites, whereas VT-TALE has two sites with D-4 and Q-5, the one less competent site with E-4, and the other site of A-4 losing the ability for hydrogen bonding. As observed in the trajectories for the distances between the inter-repeat donor and acceptor atoms, each inter-repeat hydrogen bond has a short lifetime in a range of pico-second and a changing set of the inter-repeat sites transiently form the hydrogen bonds during the structure dynamics of TALE (Fig. 3b).

### Comparing DNA-binding between CT-TALE and VT-TALE

**Figure 4:**
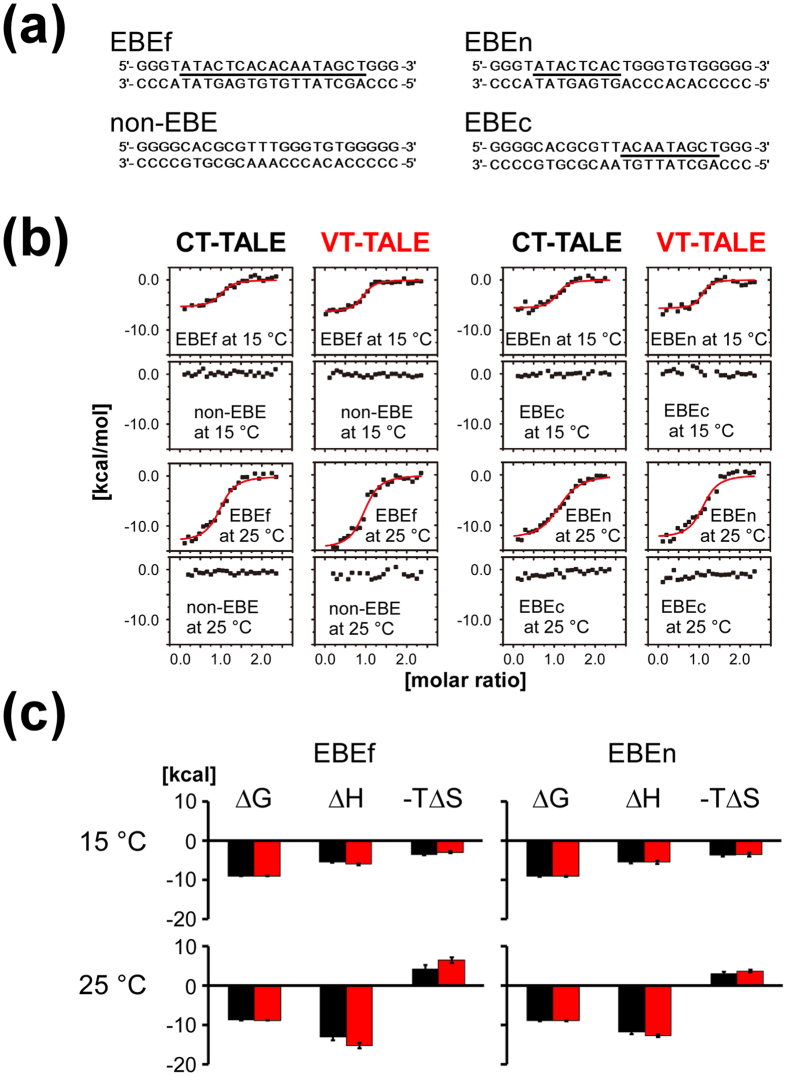
Comparison between the DNA binding mechanism of VT-TALE and CT-TALE. (**a**) The DNA sequences used in this study. EBEf (effector binding element): full target sequence; non-EBE: non-target sequence; EBEn: N-terminal half (5′ side) TAL-repeat array target sequence; EBEc: C-terminal half (3′ side) TAL-repeat array target sequence. The underlined nucleotides are target sequences recognised by our TALE samples. (**b**) ITC analyses of CT-TALE and VT-TALE against a series of DNAs. Each plot of the total heat released is presented as a function of the molar ratio of added DNA, which was subtracted by the heat generated owing to the dilution of the titrants. The red lines represent the nonlinear, least-squares best fit to the experimental data, using a one-site model. (**c**) The thermodynamic signatures of TALEs binding to EBEf (left) and EBEn (right). The upper and lower panels indicate the results at 15 and 25 °C, respectively. The results of CT-TALE and VT-TALE are presented in black and red, respectively. All experiments were carried out twice.

The binding of CT- and VT-TALEs to the target DNA sequence denoted as the effector binding element (EBE)^24^ was explored by isothermal titration calorimetry (ITC). To compare the roles of the N- and C-terminal TAL repeats in binding, we used DNAs including half of the EBE sequence, EBEn (5′ side) and EBEc (3′ side) (Fig. 4a). The DNA with the full EBE sequence is termed EBEf, and a random sequence used for reference is referred to as non-EBE (Fig. 4a). The binding of the TALEs to non-EBE was not observed under the present conditions.

The thermodynamic parameters from the ITC experiments are summarised in Table 1. The ITC experiments were performed at two different temperatures, 15 °C and 25 °C, to elucidate the change in the entropic term by increasing the temperature (Fig. 4b).

CT- and VT-TALEs had the comparable affinities to EBEf at the temperatures (Fig. 4c). Both CT- and VT-TALEs showed negative ΔS values with an increase in ΔH gain at 25 °C, resulting in reduced affinities to DNA at higher temperatures (Table 1). The ΔG values for CT- and VT-TALEs in binding to EBEn were similar to the values in their binding to EBEf at 15 °C (Fig. 4c); the differences were within the errors (Table 1). At 25 °C, the ΔG values for the CT-TALE binding to EBEf and EBEn were also similar within the errors, but the ΔG value for the VT-TALE binding to EBEn was significantly lower than that for the case to EBEf (Table 1). This finding implies the N-terminal part of the TALE mostly determines its DNA binding affinity, which is consistent with the previous report^13^. In contrast, CT- and VT-TALEs did not bind to EBEc, and the C-terminal TAL-repeats are less responsible for the DNA binding (Fig. 4b).

CT- and VT-TALEs showed similar ΔG values in binding to EBEf; however, the ΔH and ΔS values were significantly different between the TALEs at 15 °C and 25 °C (Fig. 4c). In their biding to EBEn, ΔG values for CT- and VT-TALEs were also similar to each other at the temperatures, and the differences in ΔH and ΔS values were significant at 25 °C. VT-TALE had greater gain in ΔH and more cost in ΔS relative to CT-TALE as in the case for the binding to EBEf (Fig. 4c). The observation indicates that CT- and VT-TALE have different modes of binding to DNA, and the difference is amplified in the binding to the longer target sequence at higher temperature.

VT-TALE has a significantly increased ΔH gain in binding to EBEf relative to the binding to EBEn at 25 °C, whereas CT-TALE showed statistically comparable ΔH values between the binding to EBEf and EBEn at the same temperature (Fig. 4c). The increased ΔH gain in the VT-TALE binding to EBEf implies that the greater the number of the TAL-repeats engaged in the DNA binding in VT-TALE compared with CT-TALE. The difference in the thermodynamic parameters, including ΔH and ΔS between CT- and VT-TALEs in binding to EBEn, were less significant compared with the case with EBEf at 25 °C (Fig. 4c). The gained ΔH in the binding of VT-TALE to EBEf can be ascribed to the engagement of the additional C-terminal TAL-repeats. The marginal difference in the ΔH values in the binding of CT-TALE to EBEf and EBEn indicates that the C-terminal TAL-repeats in CT-TALE are not bound to EBEf, which is a keen contrast to the binding mode of the VT-TALE.

VT-TALE has greater structural dynamics along the superhelical axis compared with CT-TALE, as experimentally suggested by the PDI values in the DLS experiments (Fig. 2c). Expanding the engagement of the TAL-repeat in the binding DNA should limit the superhelical motion of the TAL-repeat array, which explains the significantly increased entropy loss in VT-TALE in association with the increased enthalpy gain in its binding to EBEf relative to CT-TALE at 25 °C (Fig. 4c).

It is intriguing to note that the increasing temperature enhanced ΔH gain in the binding of both CT- and VT-TALEs to DNA (Fig. 4c). This finding suggests that the elevated superhelical motion by the temperature shift can make the TAL-repeat array become more compressed form to prompt the TAL-repeats to adapt in the DNA binding.

The change in the sign of ΔS by altering the temperature from 15 °C to 25 °C was remarkable for both CT- and VT-TALE binding to DNA (Fig. 4c). In contrast to the case at a higher temperature, the binding of the TALEs to DNA gained ΔS. The entropy gain at the lower temperature is presumably ascribed to the release of bound waters from DNA, which are excluded when TAL-repeats bind to the major groove of DNA. Given that the superhelical motion of TALE is reduced at the lower temperature, the entropy gain by releasing the bound waters should be apparent over the loss by limiting the superhelical motion of the TALEs upon binding to DNA.

## Discussion

**Figure 5:**
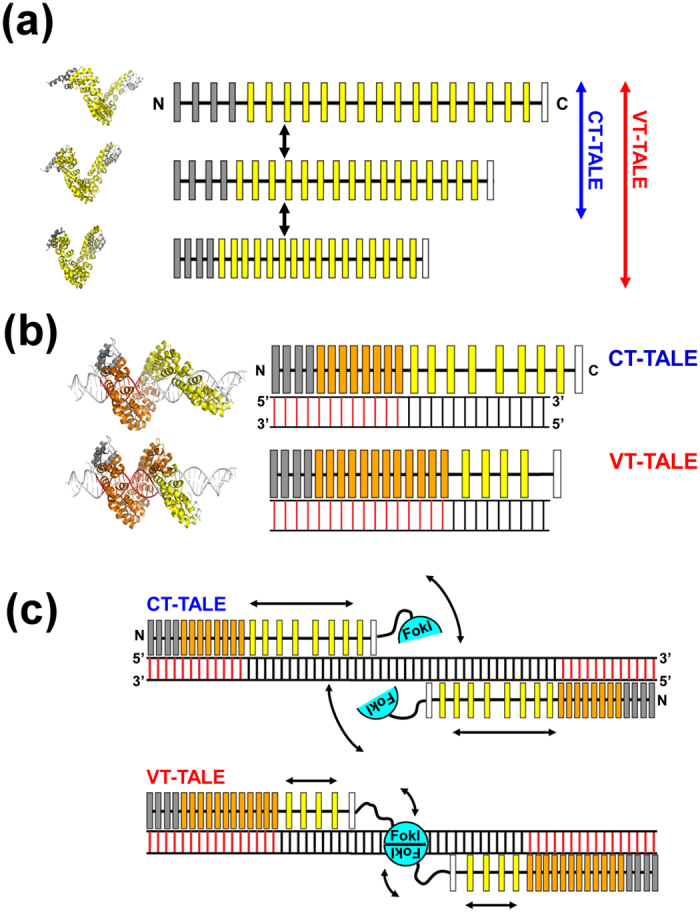
Enhanced superhelical motion by the non-RVD mutations to TALEN. (**a**) The superhelical amplitude motion of VT-TALE is greater in magnitude compared with that of CT-TALE. The model TALE structures in different conformations occurred in the molecular dynamics simulation are located on the left to the schematic drawings. The canonical TAL-repeats, N-terminal atypical repeats and C-terminal half repeats are indicated as yellow, grey and white boxes, respectively. (**b**) The number of TAL-repeats in VT-TALE (lower) that engage in DNA binding is greater than that observed for CT-TALE (upper). The DNA-bound TAL-repeats are represented by orange boxes, and the free TAL-repeats are drawn as yellow boxes. The DNA bases specifically recognised by the TAL-repeats are presented as red bars. The modelled TALE structures in the complex with DNA are located on the left of the schematic drawings for the complexes having a different number of TAL-repeats engaged in the DNA binding. (**c**) The spatial restriction of the FokI domains by the extended TAL-repeat interaction to DNA in VT-TALE (lower) could facilitate FokI dimerisation more readily than that observed for CT-TALE (upper). FokI monomers are depicted as cyan semicircles. The arrows schematically represent the magnitudes of the structural dynamics of the TAL-repeats not bound to DNA and the FokI domain associated with the C-terminal end of the TAL-repeat array.

This work aimed to explore why TALEN with the periodic non-RVD mutations exhibits increased genome editing efficacy compared with the same TALEN without such mutations^18^,^19^,^20^. We found there are different physicochemical properties between CT- and VT-TALEs as the DNA binding domain in TALEN, which are summarized as follows:

1. TALE has a superhelical motion, which is evident in several lines of experimental^14^,^15^ and theoretical^16^,^17^ evidence, which makes TALE adopt various conformations in different sizes along its superhelical axis. VT-TALE exhibited a greater magnitude in the superhelical motion and can subsequently take a wider range of structures over CT-TALE (Fig. 5a).
2. In binding to the target DNA, VT-TALE engages more TAL-repeats in the sequence recognition compared with CT-TALE (Fig. 5b).

We applied the all-atom MD simulations with a 50-ns duration to explore how the non-RVD mutations cause the above difference in the physicochemical properties between CT- and VT-TALEs. CT- and VT-TALEs in the compressed forms as found in the complex structure with DNA^14^ have become extended in 50 ns in the absence of DNA (Supplementary Fig. S4). In the MD trajectories for the extended forms (MD trajectory during 40–50 ns), we found D-4 and Q-5 in the adjacent TAL-repeats can transiently form an inter-repeat hydrogen bond through their side chain atoms (Fig. 3c). In the simulation, the non-RVD mutations changing from D-4 to A-4 or E-4 severely diminished the hydrogen bonding abilities at the mutated inter-repeat sites (Fig. 3b). Therefore, VT-TALE exhibits a reduced probability for the inter-repeat hydrogen bonding at 7.1% relative to that of CT-TALE at 12.1% (Supplementary Table S2). The inter-repeat hydrogen bonds are labile; an alternatively changing fraction of the inter-repeat sites in the TAL-repeat array can transiently form the hydrogen bonds during the structure dynamics of TALE (Supplementary Fig. S6). The reduced probability for the inter-repeat hydrogen bonds in VT-TALE could explain its reduced structural stability relative to CT-TALE (Fig. 2d).

The probability for the inter-repeat hydrogen bonding is weighted at N- and C-termini in the compressed form of CT-TALE, whereas it becomes less biased over the inter-repeat sites in the extended form (Supplementary Fig. S6). The inter-repeat hydrogen bonding probabilities at the sites comprising D-4 and Q-5 in VT-TALE are close to the values at the corresponding sites in CT-TALE in both of the forms: the pair of Q599-D632 was exceptional in both of the forms, which is presumably due to the end effect (Supplementary Fig. S6). The inter-repeat sites with non-RVD mutations, A-4 or E-4, in VT-TALE have lost or severely reduced hydrogen bonding abilities compared with the corresponding sites comprising D-4 and Q-5 in CT-TALE (Supplementary Fig. S6).

The inter-repeat sites in the extended form of CT-TALE have more equal abilities for hydrogen bonding than those in the compressed form (Supplementary Fig. S6). As noted in the inter-repeat hydrogen bonding probabilities, the compressed CT-TALE demands to break a part of the inter-repeat hydrogen bonds, as observed in the central part of the compressed form, including the residue pairs Q429-D462, Q463-D496, and Q497-D530 (Supplementary Fig. S6a). In the extended forms of CT-TALE collected from the MD trajectories in the range from 40 ns to 50 ns, the inter-repeat hydrogen bonding probability were less biased over the sites (Supplementary Fig. S6b). The present MD simulations starting from the modelled DNA bound form of the TALE (Supplementary Fig. S4) cannot sample the entire conformations that TALE experiences in solution. The inter-repeat hydrogen bonding probabilities for the sites in the extended forms, therefore, are not free from the original biased distribution forced in the DNA bound form (Supplementary Fig. S6b). Although the present MD simulation is limited in the conformational sampling, we could anticipate the extended CT-TALE should exhibit uniform probabilities for the inter-repeat hydrogen bonding over the sites. This was supported by the observed change in the probabilities for the inter-repeat hydrogen bonding between the compressed (sampled from the trajectories from 0 ns to 10 ns) and the extended forms (40–50 ns): the hydrogen bonding probabilities for Q327-D360 became lowered in the extended forms, while those for the sites including Q429-D462, Q463-D496, and Q497-D530 became increased (Supplementary Fig. S6). In considering the CT-TALE architecture comprising the TAL-repeats having the same sequence except for the RVD, it is actually hard to expect for the biased hydrogen bonding probabilities in CT-TALE in the absence of the target DNA.

In contrast to CT-TALE, half of the inter-repeat sites with A-4 or E-4 instead of D-4 in VT-TALE have lost or severely reduced hydrogen bonding abilities (Fig. 3c). It could be deduced from the difference in the inter-repeat hydrogen bonding probabilities in the compressed and extended forms that the inter-repeat hydrogen bonds stabilise the extended form, which is evident from the less biased or rather uniform formation of the inter-repeat hydrogen bonds in the extended forms (Supplementary Fig. S6. In considering the role of the inter-repeat hydrogen bonds to make the TALE preferably remain in the extended form, the mutated sites that lack the hydrogen bonds could become compressed more frequently than the sites comprising D-4 and Q-5 (Fig. 3c). VT-TALE, therefore, could have a wider range of molecular size distribution and smaller average size compared with CT-TALE, as experimentally observed (Fig. 2a and c).

TALE has to be compressed in the sequence specific binding to DNA (Supplementary Fig. S4a)^14^. In the crystal structure, all the RVDs in the TAL-repeat array interact with the target base (Supplementary Fig. S1a)^14^. In solution, however, the crafted TAL-repeats are not fully engaged in the DNA binding, but an N-terminal part of the TAL-repeats is preferentially used in the sequence recognition^13^ (Fig. 5b). TALE tends to be extended in the absence of DNA (Supplementary Fig. S4). The structural change in compressing the TAL-repeat array to make it adapted to DNA base pitch is energetically demanding. The binding of the four atypical repeats to DNA prompts the binding of the following TAL-repeats to DNA, but this cooperative effect is not propagated to the entire repeats^13^. In fact, as demonstrated in this work, the C-terminal half of the TALE did not bind to DNA even in the presence of the four atypical repeats (Fig. 4b).

Our ITC results demonstrated that VT-TALE has more TAL-repeats engaged in the sequence recognition than CT-TALE (Fig. 4c). VT-TALE has greater ΔH gain in binding to EBEf compared to CT-TALE at 25 °C. Because the difference in ΔH value was much smaller between CT- and VT-TALEs in binding to EBEn at 25 °C (Fig. 4c), the elevated ΔH gain in the VT-TALE binding to EBEf can be ascribed to the engagement of the additional C-terminal TAL-repeats, whose counterparts in CT-TALE are not bound to DNA (Fig. 5b).

It is noticeable that the increasing temperature enhanced ΔH gain for the CT- and VT-TALE binding to EBEf (Fig. 4c), which indicates that more TAL-repeats bind to the target bases at higher temperature. This finding suggests the TALE superhelical motion with a greater amplitude at higher temperature can generate the more compressed forms to engage a greater number of TAL-repeats in the sequence-specific binding (Fig. 5a and b), which consistently explains the increased entropy costs at the higher temperature (Fig. 4c).

In summing up the role of the TALE structure dynamics in its DNA binding, TALE needs to become more compressed to enable a wider range of sequence specific interactions by the TAL-repeats, which requires a lager magnitude of super helical motion. In fact, raising the temperature to activate the TALE superhelical motion can increase the number of TAL-repeats engaged in the binding by generating the compressed forms. The lack of a part of the inter-repeat hydrogen bonds in VT-TALE could allow the TAL-repeat array to sample a wider range of conformations and, thus, to become more compressed forms compared with CT-TALE, which prompts VT-TALE to have a greater number of TAL-repeats engaged in DNA binding than that in CT-TALE (Fig. 5b).

In considering the genome editing process by TALEN^10^,^11^, the stringent sequence recognition by a pair of TALEN molecules and the efficient dimerization of FokI nucleases on DNA are essential for increasing the efficacy. The stringent sequence recognition by TALEN is attained by engaging a greater number of TAL-repeats in the binding to the target bases (Fig. 5c). Given the lack of a part of inter-repeat hydrogen bonds, VT-TALE becomes more compressed forms and enables a wider range of sequence recognition by the TAL-repeats compared with CT-TALE (Fig. 5b). The TALEN with VT-TALE as its DNA binding domain is advantageous in achieving the stringent sequence recognition compared with CT-TALE.

In TALE binding to DNA, the engagement of a greater number of TAL-repeats in DNA binding is associated with increased entropy cost by reducing the superhelical motion of the TAL-repeat array. This notion was obviously demonstrated by the present ITC experiments for comparing the CT- and VT-TALE binding with EBEf at 25 °C (Fig. 4c); the increased ΔH gain in the VT-TALE binding to DNA was diminished by the greater ΔS cost, resulting in the comparable ΔG value and a DNA binding affinity similar to that of CT-TALE (Fig. 4c). This thermodynamic trade-off between the sequence specific binding and the entropy cost by the loss of the superhelical motion of the TAL-repeats is the characteristic property in the TALE binding to DNA. The amplified superhelical motion of the TAL-repeat array can increase the sequence specificity by making a greater number of TAL-repeats bound to the target base through generating the more compressed forms of the TAL-repeat array (Fig. 5a and b), while the increase in the number of bound TAL-repeats will result in the greater entropy cost by reducing the superhelical motion of the TALE.

This thermodynamic trade-off has a role in facilitating the dimerization of FokI on DNA. The greater number of TAL-repeat binding to DNA will limit the dynamics of the C-terminal TAL-repeats otherwise they are unbound to DNA and remain dynamic (Fig. 5c). The FokI is anchored to the C-terminal end of the TAL-repeat array. The diminished motion of the C-terminal TAL-repeat array, therefore, will restrict the spatial allocation of the FokI domain (Fig. 5c). Limiting the spatial allocation of FokI domains should promote the encounter probability for the FokI domains to dimerize on the target site. The increase in the sequence specificity with the binding of a greater number of the TAL-repeats, therefore, leads to enhance the FokI dimerization (Fig. 5c). TALE can co-ordinately accomplish the stringent sequence recognition and the dimerization of the FokI domains with the elaborate array structure and its associated superhelical motion (Fig. 5c).

In summary, VT-TALE engages a greater number of the TAL-repeats in the DNA binding compared with CT-TALE (Fig. 5b) due to the wider range of allowed conformations of the TAL-repeat array along the superhelical axis in VT-TALE (Fig. 3c). TALEN with VT-TALE gains increased sequence specificity and the improved efficiency for FokI dimerization (Fig. 5c), which explains its increased genome editing efficacy compared with the TALEN with CT-TALE.

Finally, we add the comments on the mutation sites in the TAL-repeat. In this work, we described how the non-RVD mutations change the structure and dynamics of TALE, and the role of the residues D-4 and Q-5 in the neighbouring repeats were emphasized. The non-RVD residues are D-4 and D-32 in each repeat, but the significance of the mutation to D-32 was not clear. Instead, the role of the Q-5 as the partner to make the inter-repeat hydrogen bonds with D-4 was noted. Mutations to Q-5 in the TAL-repeats would modify the TALE binding ability to DNA.

## Methods

### Sample preparations

The cDNAs encoding dAvrBs3 TALEs with and without the periodically mutated repeats were prepared according to previous reports^20^,^25^,^26^. Either type of the dAvrBs3 gene was cloned into pET28a using *Nde*I and *Eco*RI sites and transformed into *E. coli* Rosetta (DE3). Cells were grown in LB broth at 37 °C to an OD_600_ = 0.7, and isopropyl β-d-thiogalactopyranoside was added to a final concentration of 0.5 mM to induce protein expression. The culture was incubated for a further 20 h at 16 °C. Cells were collected by centrifugation and resuspended in buffer A (50 mM Tris-HCl (pH 8.0), 200 mM NaCl, 20 mM imidazole, 14 mM 2-mercaptoethanol, 20% glycerol) and then subjected to sonication. The supernatant of the resultant lysate was applied to a HisTrap column (GE Healthcare, Waukesha, WI, USA) equilibrated with buffer A. The column was washed with 200 ml of buffer A followed by 100 ml of high salt buffer containing 4 M NaCl in buffer A to remove the contaminated nucleic acids and finally washed with 100 ml of buffer A. The removal of the contaminating DNAs from the sample solution was checked by Hoechst 33342 dye (Doujindo Laboratories, Tokyo, Japan). The protein was eluted by buffer A containing 500 mM imidazole, and the sample solution was extensively dialysed against buffer B (50 mM Tris-HCl (pH 8.0), 200 mM NaCl, 14 mM 2-mercaptoethanol, 5% glycerol) at 4 °C. The dialysed sample solution was applied to a HiTrap Heparin column (GE Healthcare) equilibrated with buffer B. The protein was further purified with a NaCl gradient from 0.2 to 1.0 M in buffer B. The collected proteins were further purified by SEC (HiLoad 26/600 Superdex 200 pg, GE Healthcare) with a buffer solution containing 50 mM Tris-HCl (pH 8.0), 200 mM NaCl, 1 mM dithiothreitol (DTT) and 5% glycerol. The purified protein includes a His_6_-tag at the N-terminus.

### Molecular size estimation by SEC

The Stokes radius (*R*_S_) of each TALE was estimated using the elution time with reference to a standard globular protein set (GE Healthcare)^27^. The experimental conditions used for the chromatography were the same as described in the sample preparation. The HiLoad 26/600 Superdex 200 pg column was calibrated with a Gel Filtration Protein Calibration Kit (GE Healthcare) at a linear flow rate of 1 ml/min. The protein standards were as follows: Aprotinin (*M*_W_: 6.5 kDa, *R*_S_: 13 Å), Ribonuclease A (*M*_W_: 13.7 kDa, *R*_S_: 16 Å), Carbonic anhydrase (*M*_W_: 29.0 kDa, *R*_S_: 20 Å), Ovalbumin (*M*_W_: 44.0 kDa, *R*_S_: 30 Å), and Conalbumin (*M*_W_: 75.0 kDa, *R*_S_: 36 Å)^27^. The gel-phase distribution coefficient, *K*_*av*_, for each protein was calculated using the equation *K*_*av*_ = (*V*_*e*_ − *V*_0_)/(*V*_*c*_ − *V*_0_), where *V*_*e*_ is the elution volume for the protein, *V*_*0*_ is the column void volume determined using blue dextran 2000 (GE Healthcare) monitored at a wavelength of 280 nm, and *V*_*c*_ is the geometric column volume (320 ml). Plots of 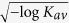 vs. *R*_S_ were used to determine the Stokes radii of proteins. The minimal radius of a globular protein (*R*_min_) for a given molecular weight (*M*_w_) was estimated according to the relation^21^, 
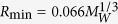
.

### Circular dichroism (CD) spectroscopy

The CD spectra of the TALEs were measured on a JASCO (Tokyo, Japan) J-720W spectrometer at 25 °C using a 1 mm quartz cell. The protein concentrations were adjusted to 3 μM in a buffer solution containing 50 mM Tris-HCl (pH 8.0), 200 mM NaCl, 1 mM DTT and 5% glycerol. CD data were measured from 190 to 260 nm at a scanning rate of 100 nm/min with a single scan. The CD data were presented in terms of ellipticity, *θ* (degrees), which were normalised by scaling to molar concentrations of the repeating unit of a polymer. The molar ellipticity is described as


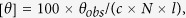


where *θ*_*obs*_ is the observed ellipticity in degree, *c* is the concentration of protein in molar, *N* is the number of amino acids in the protein, and *l* is the cell path length in cm.

### DLS

DLS measurements were performed with a Malvern Zetasizer ZS (Malvern Instruments Ltd, Worcs, UK) at 25 °C. TALEs were dissolved in 50 mM Tris-HCl buffer (pH 8.0), 200 mM NaCl and 5% glycerol, and the protein concentration was adjusted to 5 μM. Measurement of each sample was repeated five times to evaluate experimental uncertainties.

### DSC

DSC thermograms were collected by a VP-capillary DSC platform (Microcal LLC, Northampton, MA, USA) up to 95 °C at scan rates of 1.0 °C/min. For the measurements, the protein concentrations were adjusted to 1.4 mg/ml in 50 mM Tris-HCl (pH 8.0), 200 mM NaCl, 1 mM DTT and 5% glycerol.

### ITC

ITC measurements were performed at 15 °C and 25 °C on a Microcal Auto-iTC200 calorimeter (GE Healthcare) using the four types of double-stranded DNAs (Fig. 4a). TALEs and DNAs were dissolved in 50 mM Tris-HCl (pH 8.0), 200 mM NaCl, 1 mM DTT and 5% glycerol. The experiments consisted of a series of 1.5 μl injections of 96 μM DNA into 200 μl TALE protein solution in the thermostatic cell with an initial delay of 60 s, a 3 s duration of injection and a spacing between injections of 150 s. The protein concentrations were determined by the absorption at 280 nm using a molar extinction coefficient of 14.98 mM^−1^cm^−1^. All concentrations were measured on a Nanodrop 2000 (Thermo Fisher Scientific, Massachusetts, USA). The DNA compounds were purchased from Hokkaido System Science Co. Ltd. (Sapporo, Japan). Each oligonucleotide was dissolved in water (1 mL, 3 mg/mL). After thoroughly mixing a pair of DNA solutions, the solution was incubated at greater than 85 °C for 10 min using Thermolyne 17600 (Barnstead) and then slowly cooled to room temperature. The quality of each double-strand DNA was over 90% as assessed by analytical HPLC on LabSolutions LC-System (Shimazu) by using an ENDURO C18G (250 mm × 4.6 mm, 5 μm) column (SGE Analytical Science) with a gradient of 0 to 100% acetonitrile in 20 mM triethylammonium acetate (pH 7.0) solution for 30 min at 1 mL/min. The final DNA concentrations were determined by A_260_ using SpectraMax i3 plate reader (Molecular Devices). The corrected binding isotherms were fitted using a single-site model. The collected data were analysed with the Microcal ORIGIN software (GE Healthcare). For the purpose of the error evaluation, each measurement was repeated thrice. The average values and standard deviations for thermodynamic parameters are listed in Table 1.

### MD simulations

All-atom models of VT- and CT-TALE were constructed from the DNA-bound dHax3 TALE structure (PDB ID: 3V6T^14^, chain A) by substituting atoms involved in the mutations according to the procedure described in the Supplementary Text. To consider structural changes after DNA unbinding, these models were simulated without DNA in explicit water with 0.15 mM of KCl. NAMD 2.9^28^ and VMD 1.9.1^29^ were used in the modelling, simulation and analysis. Details in the simulations are provided in the Supplementary Information.

## Additional Information

**How to cite this article**: Tochio, N. *et al*. Non-RVD mutations that enhance the dynamics of the TAL repeat array along the superhelical axis improve TALEN genome editing efficacy. *Sci. Rep.* **6**, 37887; doi: 10.1038/srep37887 (2016).

**Publisher's note:** Springer Nature remains neutral with regard to jurisdictional claims in published maps and institutional affiliations.

## Supplementary Material

Supplementary Information

## Figures and Tables

**Table 1:** ITC-derived thermodynamic parameters of the binding of TALE to DNA.

DNA^a^	Temp. (°C)	TALE	Thermodynamic parameters^b^				
			Sites (n)	ΔG (kcal/mol)	ΔH (kcal/mol)	−TΔS (kcal/mol)	K_D_ (nM)
EBEf	15	CT-TALE	0.94 ± 0.05	−9.02 ± 0.17	−5.42 ± 0.26	−3.60 ± 0.31	138.89 ± 40.81
		VT-TALE	0.88 ± 0.05	−9.06 ± 0.07	−6.05 ± 0.21	−3.02 ± 0.22	131.33 ± 16.20
	25	CT-TALE	0.99 ± 0.01	−8.80 ± 0.15	−13.01 ± 0.92	4.22 ± 0.93	345.98 ± 80.94
		VT-TALE	0.93 ± 0.02	−8.84 ± 0.06	−15.23 ± 0.71	6.39 ± 0.71	325.73 ± 34.33
EBEn	15	CT-TALE	1.03 ± 0.03	−9.17 ± 0.09	−5.47 ± 0.38	−3.70 ± 0.39	108.90 ± 16.52
		VT-TALE	0.99 ± 0.04	−9.11 ± 0.21	−5.54 ± 0.42	−3.57 ± 0.47	117.03 ± 46.07
	25	CT-TALE	1.13 ± 0.03	−8.87 ± 0.17	−11.87 ± 0.49	3.00 ± 0.52	305.31 ± 88.05
		VT-TALE	1.12 ± 0.02	−8.97 ± 0.07	−12.66 ± 0.33	3.69 ± 0.34	261.85 ± 31.04

^a^EBE represents ‘effector binding element’. EBEf contains the full recognition sequence, whereas EBEn has the sequence recognised by the N-terminal eight TAL-repeats. The other types of DNA used in this work (Fig. 4a) did not give any thermograph responses; thus, they are not listed in this table.

^b^Each measurement was repeated thrice.
