# Supplementary material for: Non-RVD mutations that enhance the dynamics of the TAL repeat array along the superhelical axis improve TALEN genome editing efficacy
Source: Sci Rep. 2016 Nov 24;6:37887. doi: 10.1038/srep37887 (PMC5121632; doi:10.1038/srep37887)
Supplement: Supplementary Information [file srep37887-s1.pdf]

Supplementary Information

Non-RVD mutations that enhance the dynamics of the TAL repeat array along the superhelical axis improve TALEN genome editing efficacy

Naoya Tochio[1,a], Kohei Umehara[2,a], Jun-ichi Uewaki[1,a], Holger Flechsig[2], Masaharu Kondo[3], Takehisa Dewa[3], Tetsushi Sakuma[2], Takashi Yamamoto[2], Takashi Saitoh[4], Yuichi Togashi[1] and Shin-ichi Tate[1,2,*]

[1]Research Center for the Mathematics on Chromatin Live Dynamics (RcMcD), Hiroshima University, 1-3-1 Kagamiyama, Higashi-Hiroshima 739-8526, Japan

[2]Department of Mathematical and Life Sciences, Graduate School of Science, Hiroshima University, 1-3-1 Kagamiyama, Higashi-Hiroshima 739-8526, Japan

[3]Department of Life Science and Applied Chemistry, Nagoya Institute of Technology, Gokiso-cho, Showa-ku, Nagoya 466-8555, Japan

[4]Faculty of Pharmaceutical Sciences and Center for Research and Education on Drug Discovery, Hokkaido University, Kita-ku, Sapporo 060-0812, Japan

[a]These authors equally contributed to this work.

Supplementary Text

Modelling CT- and VT-TALE structures

CT- and VT-TALE structures used in the all-atom MD simulations were built from the chain A of the DNA-bound dHax3 TALE crystal structure (PDB ID: 3V6T) by changing the amino acid residues at positions 4 and 32 as in CT- and VT-TALEs, respectively (Supplementary Fig. S2). In modelling the structures, we used VMD 1.9.1[1] and the Mutator 1.3 plugin to substitute atoms in the residues at mutation sites, and subsequently applied Automatic PSF builder to supplement missing atoms. CT- and VT-TALE used in the experiments comprise four units, with each unit composed of four TAL-repeats, and they have 16.5 TAL-repeats in total (Fig. 1c). The modelled CT- and VT-TALE in the simulations, however, contain only 11.5 TAL-repeats, because the dHax3 TALE crystal structure used as the template for modelling has 11.5 TAL-repeats. In the analyses for the inter-repeat hydrogen bonding using the MD trajectories, we considered the 11.5 repeats (Supplementary Fig. S2). It should be noted that the last TAL-repeat in the model TALEs were built from the half-repeat in dHax3, having the different sequence for the residues 16–34 from the corresponding regions of the other TAL-repeats (Supplementary Fig. S2). The sequence and the spatial structure of the N-terminal half of the last half-repeat were the same as the parts in the other TAL-repeats. Therefore, the N-terminal segment of the last TAL-repeat (the 12th repeat) was also considered in the analyses for the inter-repeat hydrogen bonding.

MD simulation of the TALEs starting from the compressed DNA bound form

The MD simulations for the CT- and VT-TALEs in the absence of DNA were carried out starting from the compressed structures in the complex with DNA, which mimicked their unbinding process from DNA. We used the models generated from the crystal structure of dHax3, as described above. The modelled TALEs were soaked into a 90 × 90 × 150 Å TIP3P explicit water box with 0.15 mol/L of KCl added. The CHARMM36 force field and NAMD 2.9 program[2] were used, with the NPT ensemble (300 K, 1 atm, Langevin), particle-mesh Ewald (cutoff at 12 Å is applied, also for van der Waals interactions with

switching at 10 Å) and a 2 fs time-step with SHAKE/SETTLE. After energy minimisation, the MD simulations were carried out for 50ns, in triplicate for each of the TALEs.

To monitor the conformational changes, the distance between α-carbon atoms of residues 303 and 675 was measured (Supplementary Fig. S3b). These residues are separated by ca. 1 turn around the DNA; the distance is 35.76 Å in the original DNA-bound crystal structure, and 60.54 Å in the DNA-free crystal structure (PDB ID: 3V6P). The Root-Mean-Square Displacement (RMSD) to the initial (DNA-bound) and DNA-free crystal structures were also calculated (Supplementary Fig. S3c and S3d). In the calculation of RMSD, the backbone atoms (C, C^{α}, and N) in only those residues common to both crystal structures (residues 303 to 675) were considered (except undetermined coordinates for Arg448 in 3V6P), and global translation and rotation were cancelled. Both the CT- and VT-TALE models significantly elongated during the 50 ns simulation.

The inter-repeat hydrogen bonds transiently formed during the dynamics of CT- and VT-TALEs

In exploring the inter-repeat hydrogen bonding efficiencies in CT- and VT-TALEs, we considered all the possible donor-acceptor pairs between the 5th residue in each TAL-repeat (residues 293 to 633) and the 4th residue in the next repeat (residues 326 to 666). The hydrogen bond is counted if the donor-acceptor distance is less than 3 Å and the donor-hydrogen-acceptor angle is less than 20 degrees. To capture the labile hydrogen bonding, we sampled the structure every 1 ps over the designated period. Probability of forming a hydrogen bond for each considered pair of residues was calculated, and then averaged (Supplementary Table S1). Although we considered all the possible donor-acceptor pairs, almost all the hydrogen bonds were between the sidechains, and mostly between $N^{\varepsilon 2}$-$H^{\varepsilon 2}$ of Q-5 and O^{δ} of D-4 (or O^{ε} of E-4). The contribution of the back bone atoms was found ignorable (the hydrogen bonding probability was less than 10^{-5}). Ala-4 has no chance to form the hydrogen bonds (probability $< 10^{-5}$ observed). In addition, E-4 in VT-TALE formed inter-repeat hydrogen bonds with much lower (ca. 1/20) efficiency than D-4. The overall probability of inter-repeat hydrogen bonding was significantly decreased in VT-TALE, due to the mutations of D-4 to A-4 or E-4.

As seen in the MD trajectories, each inter-repeat hydrogen bond is labile and has a short life time in a range of ps (Fig. 3b). The probability of the hydrogen bonding represents the number of the inter-repeat connections allowed to be formed simultaneously at a moment. The inter-repeat hydrogen bond can be formed at any inter-repeat position in the TAL-repeat array without any preference to specific sites; the low hydrogen bonding probability implies that all the inter-repeat hydrogen bonds cannot be formed simultaneously, but only a probable fraction of the selected pairs are allowed to form hydrogen bonds.

To show the dynamic engagement of atoms in the hydrogen bonding, we monitored the distances between O^{δ} of Asp-4 (or O^{ε} of Glu-4) and $H^{\varepsilon 2}$ of Gln-5 in the neighbouring repeats in the MD trajectories, which were sampled at every 10 ps (Fig. 3b, using the trajectory of Trial 1 in Supplementary Fig. S3b to S3d). At each inter-repeat site, there are four possible atomic pairs in forming the inter-repeat hydrogen bonds (two O^{δ}/O^{ε} acceptor atoms in Asp-4/Glu-4 by two $H^{\varepsilon 2}$ atoms in Gln-5); each atom pair is shown in different colours (Fig. 3b).

REFERENCES

1 Humphrey, W., Dalke, A. & Schulten, K. VMD: Visual molecular dynamics. *Journal of Molecular Graphics* **14**, 33-38, doi:10.1016/0263-7855(96)00018-5 (1996).

2 Phillips, J. C. *et al.* Scalable molecular dynamics with NAMD. *Journal of Computational Chemistry* **26**, 1781-1802, doi:10.1002/jcc.20289 (2005).

Acknowledgements

NAMD and VMD were developed by the Theoretical and Computational Biophysics Group at the Beckman Institute for Advanced Science and Technology at the University of Illinois, Urbana-Champaign.

Supplementary Figure Legends

Supplementary Figure S1. Superhelical axis for TALE protein. (a) The crystal structure of dHax3 in the complex with double stranded DNA (PDB ID: 3V6T). The dotted line is defined as the superhelical axis of the DNA. (b) The DNA bound form dHax3 shown in hiding the DNA structure (PDB ID: 3V6T); the dotted line is the superhelical axis for the TALE. (c) The extended dHax3 crystal structure in the absence of DNA (PDB ID: 3V6P) with the dotted line as the superhelical axis for the TALE.

Supplementary Figure S2. Structural instabilities for the TALE proteins were estimated by the CD experiment. The CD spectra of the TALEs were measured on a JASCO (Tokyo, Japan) J-720W spectrometer from 25°C to 80°C using a 1 mm quartz cell. The protein concentrations were adjusted to 3 μM in a buffer solution containing 50 mM Tris-HCl (pH 8.0), 200 mM NaCl, 1 mM DTT and 5% glycerol. The CD data were presented in terms of the ratio of the molar ellipticity at 222 nm as the loss of the structure, which is given by:

$$[Loss\ of\ \ the\ structute,\%] = \frac{\theta_{222,obs} - \theta_{222,\min}}{\theta_{222,\max} - \theta_{222,\min}} \times 100$$

where $\theta_{222,\mathrm{obs}}$ is the observed ellipticity at the corresponding temperature, $\theta_{222,\mathrm{max}}$ and $\theta_{222,\mathrm{min}}$ are the maximum and minimum molar ellipticity values at 222 nm at the temperature range from 25 and 80 ºC, respectively. The overlaid presentation of the CD spectral changes by heating the samples are shown: CT-TALE (blue) and VT-TALE (red).

Supplementary Figure S3. The amino acid sequence in the modelled CT- and VT-TALEs used in the MD simulation. The sequence comes from dHax3 crystal structure (PDB ID: 3V6T) used as the template for the models. The residues marked in filled boxes at positions 4 and 32 are changed to generate the models for CT- and VT-TALEs.

Supplementary Figure S4. The TALE repeat elongates in the absence of the target DNA. (a) The positions of the markers (α-carbons of residues 303 and 675) in the DNA-bound and DNA-free crystal structures of TALE. (b) Time series of the distance between the markers observed in the MD trajectory sampled from 0 to 50 ns at every 10 ps. Simulations were done in triplicate for each of the TALEs. The trajectories are displayed by different line patterns in blue (CT-TALE) and red (VT-TALE). (c,d) Time series of the RMSD to (c) the initial DNA-bound and (d) the DNA-free crystal structures, calculated from the same trajectories as in panel (b); sampling intervals and notation (line colours and patterns) are also the same. To allow comparison between panels (c) and (d), we considered only residues 303 to 675, common to the two crystal structures (see Supplementary Text for detail).

Supplementary Figure S5. Snapshots of the CT- and VT-TALE models in the compressed forms. Snapshots of the CT- and VT-TALE models in the compressed forms. The structures are viewed from the C-terminal side of the superhelical structure and its 90-degree rotated position. For the four TAL-repeats in the centre of the array, the side chains of the residues positioned at 4 and 5 in each repeat of CT- and VT-TALEs are drawn as ball and stick representations. The residues at position 4, a non-RVD residue, in each TAL-repeat are marked in purple. The snapshots are taken after 5 ns of equilibration with weak harmonic restraint (stiffness 1 pN/Å) to each α-carbon atom at the original position.

Supplementary Figure S6. The probability of forming inter-repeat hydrogen bonds for each pair of residues. (a) in the compressed form and (b) in the extended form. The samples and criteria are the same as in Supplementary Table S1.

Supplementary Table S1. Denaturation temperature, calorimetric and van’t Hoff enthalpies for the unfolding process of CT-TALE and VT-TALE.

	T_m (ºC)	ΔHº (kJ/mol)	ΔH_{vH}
CT-TALE	61.71 ± 0.02	62.9 ± 1.4	2,490 ± 30
	63.41 ± 0.01	101.0 ± 2.0	2,460 ± 50
VT-TALE	52.07 ± 0.01	194.0 ± 1.0	1,830 ± 10

Supplementary Table S2. The probability of forming inter-repeat hydrogen bonds in the compressed and extended forms of CT- and VT-TALEs. [a]The inter-repeat hydrogen bonding probability was calculated from the structures in the MD trajectories, sampled every 1 ps during 0 ns – 10 ns (compressed form) and 40 ns – 50 ns (extended form), 3 trials each. The hydrogen bond is counted if the donor-acceptor distance is less than 3 Å and the donor-hydrogen-acceptor angle is less than 20 degrees; all the possible donor-acceptor pairs between the 5th residue in each repeat (residues 293 to 633) and the 4th residue in the next repeat (residues 326 to 666) were considered. Note that most hydrogen bonds were between the sidechains, and the contribution of the backbone atoms was negligible (bonding probability < 10^{-5}). [b]The CT-TALE model has 11 Asp residues at the 4th positions, while the VT-TALE model has 5 Asp (D), 3 Glu (E) and 3 Ala (A) residues there. The overall probability is equal to the average of probability weighted by the numbers of inter-repeat residue-pairs.

TALE	MD trajectory	Inter-repeat pair	Probability[a]	Overall Probability[b]
CT	0 – 10 ns	D-4 and Q-5	0.119	0.119
	40 – 50 ns	D-4 and Q-5	0.121	0.121
VT	0 – 10 ns	D-4 and Q-5	0.119	0.056
		E-4 and Q-5	0.006	
		A-4 and Q-5	0.000	
	40 – 50 ns	D-4 and Q-5	0.152	0.071
		E-4 and Q-5	0.007	
		A-4 and Q-5	0.000	

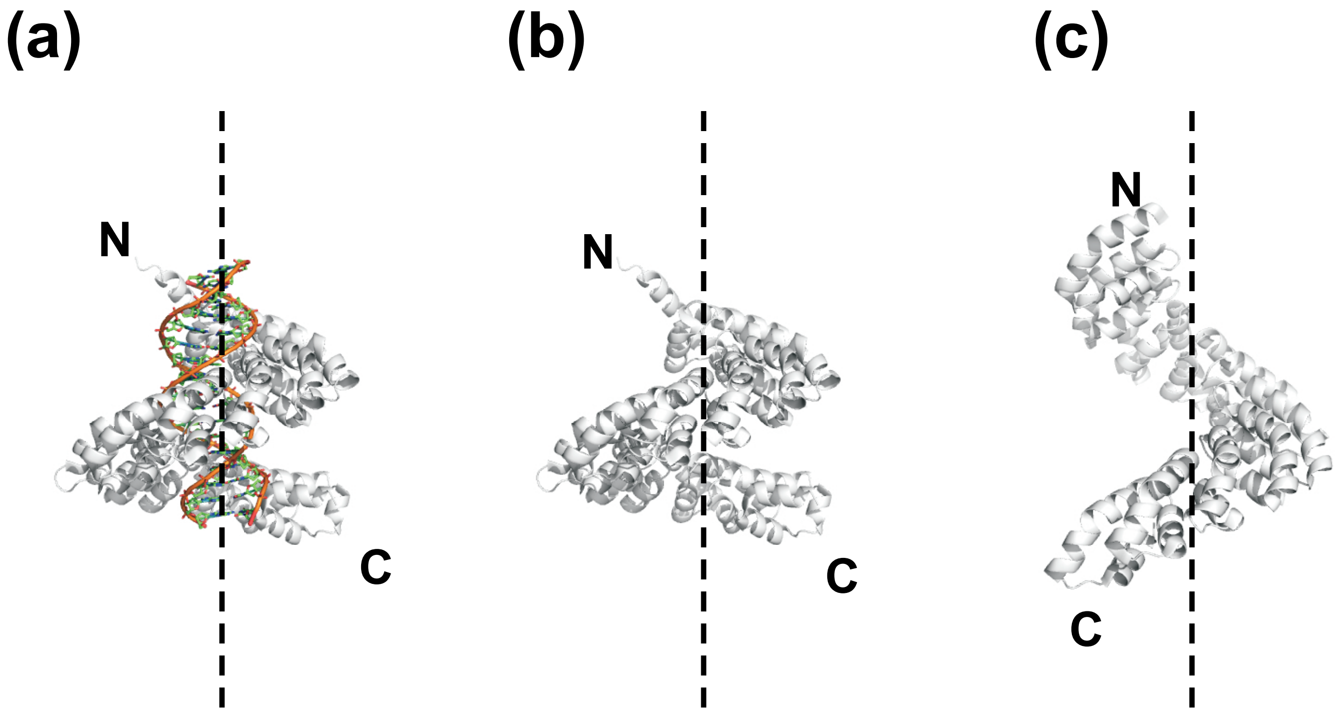

Fig. S1

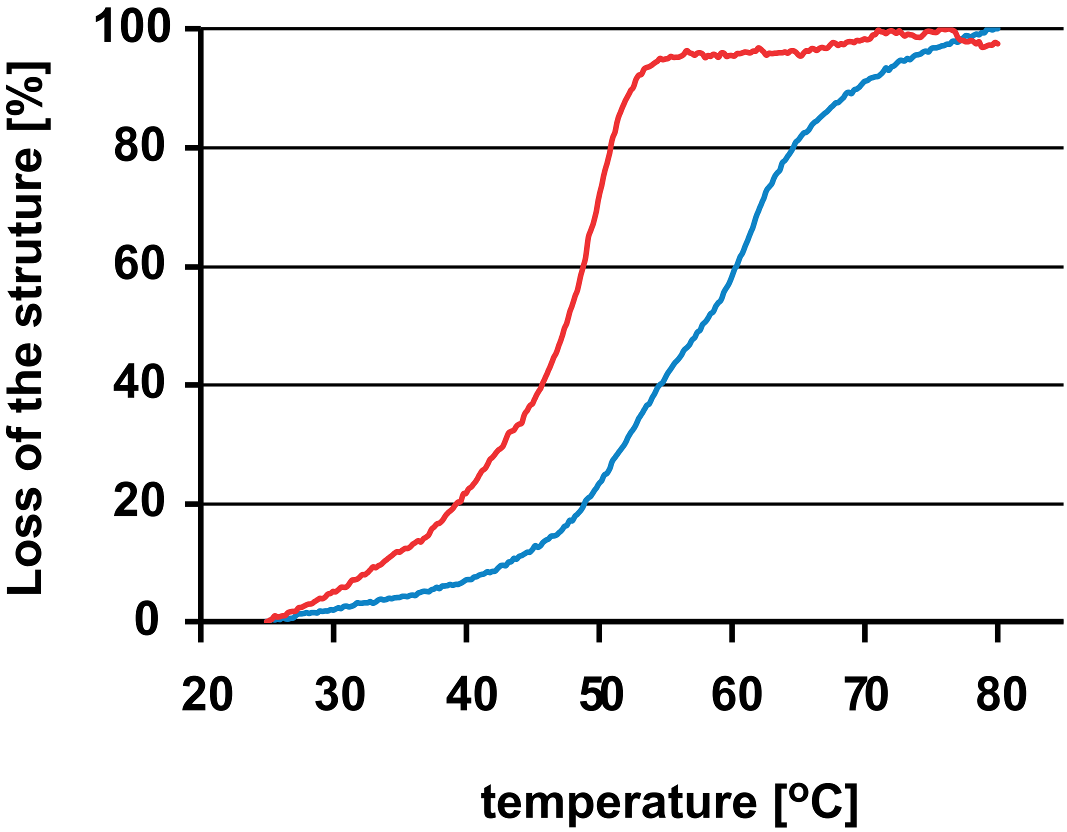

100
80
60
40
20
0
Loss of the struture [%]
20
30
40
50
60
70
80
temperature [°C]

Fig. S2

		1	2	3	4	5	6	7	8	9	10	11	12	13	14	15	16	17	18	19	20	21	22	23	24	25	26	27	28	29	30	31	32	33	34	
Position N-tail	231	-	-	-	-	-	-	-	-	-	-	Q	W	S	G	A	R	A	L	E	A	L	L	T	V	A	G	E	L	R	G	P	P	L	Q	254
Pseudo repeat	255	L	D	T	G	Q	L	L	K	I	A	K	R	G	G	V	T	A	V	E	A	V	H	A	W	R	N	A	L	T	G	A	P	L	N	288
TAL-repeat-1	289	L	T	P	E	Q	V	V	A	I	A	S	H	D	G	G	K	Q	A	L	E	T	V	Q	R	L	L	P	V	L	C	Q	A	H	G	322
	CT-TALE				D																												D			
	VT-TALE				D																												D			
TAL-repeat-2	323	L	T	P	Q	Q	V	V	A	I	A	S	H	D	G	G	K	Q	A	L	E	T	V	Q	R	L	L	P	V	L	C	Q	A	H	G	356
	CT-TALE				D																												D			
	VT-TALE				E																															
TAL-repeat-3	357	L	T	P	E	Q	V	V	A	I	A	S	H	D	G	G	K	Q	A	L	E	T	V	Q	A	L	L	P	V	L	C	Q	A	H	G	390
	CT-TALE				D																												D			
	VT-TALE				D																															
TAL-repeat-4	391	L	T	P	E	Q	V	V	A	I	A	S	N	G	G	G	K	Q	A	L	E	T	V	Q	R	L	L	P	V	L	C	Q	A	H	G	424
	CT-TALE				D																												D			
	VT-TALE				A																												D			
TAL-repeat-5	425	L	T	P	Q	Q	V	V	A	I	A	S	N	G	G	G	K	Q	A	L	E	T	V	Q	R	L	L	P	V	L	C	Q	A	H	G	458
	CT-TALE				D																												D			
	VT-TALE				D																												D			
TAL-repeat-6	459	L	T	P	Q	Q	V	V	A	I	A	S	N	G	G	G	K	Q	A	L	E	T	V	Q	R	L	L	P	V	L	C	Q	A	H	G	492
	CT-TALE				D																												D			
	VT-TALE				E																															
TAL-repeat-7	493	L	T	P	Q	Q	V	V	A	I	A	S	N	S	G	G	K	Q	A	L	E	T	V	Q	R	L	L	P	V	L	C	Q	A	H	G	526
	CT-TALE				D																												D			
	VT-TALE				D																															
TAL-repeat-8	527	L	T	P	Q	Q	V	V	A	I	A	S	N	G	G	G	K	Q	A	L	E	T	V	Q	R	L	L	P	V	L	C	Q	A	H	G	560
	CT-TALE				D																												D			
	VT-TALE				A																												D			
TAL-repeat-9	561	L	T	P	Q	Q	V	V	A	I	A	S	H	D	G	G	K	Q	A	L	E	T	V	Q	R	L	L	P	V	L	C	Q	A	H	G	594
	CT-TALE				D																												D			
	VT-TALE				D																												D			
TAL-repeat-10	595	L	T	P	E	Q	V	V	A	I	A	S	N	G	G	G	K	Q	A	L	E	T	V	Q	R	L	L	P	V	L	C	Q	A	H	G	628
	CT-TALE				D																												D			
	VT-TALE				E																															
TAL-repeat-11	629	L	T	P	E	Q	V	V	A	I	A	S	H	D	G	G	K	Q	A	L	E	T	V	Q	R	L	L	P	V	L	C	Q	A	H	G	662
	CT-TALE				D																												D			
	VT-TALE				D																															
Half-repeat + C-tail	663	L	T	P	Q	Q	V	V	A	I	A	S	N	G	G	G	R	P	A	L	E	S	I	V	A	Q	L	S	R	P	D	P	A	L	A	696
	CT-TALE				D																												D			
	VT-TALE				A																												D			
C-tail	697	A	L	T	N	D	H	L	V	A	L	A	C	L	G	G	R	P	A	L	D	A	V	K	K	L	-	-	-	-	-	-	-	-	-	721

Fig. S3

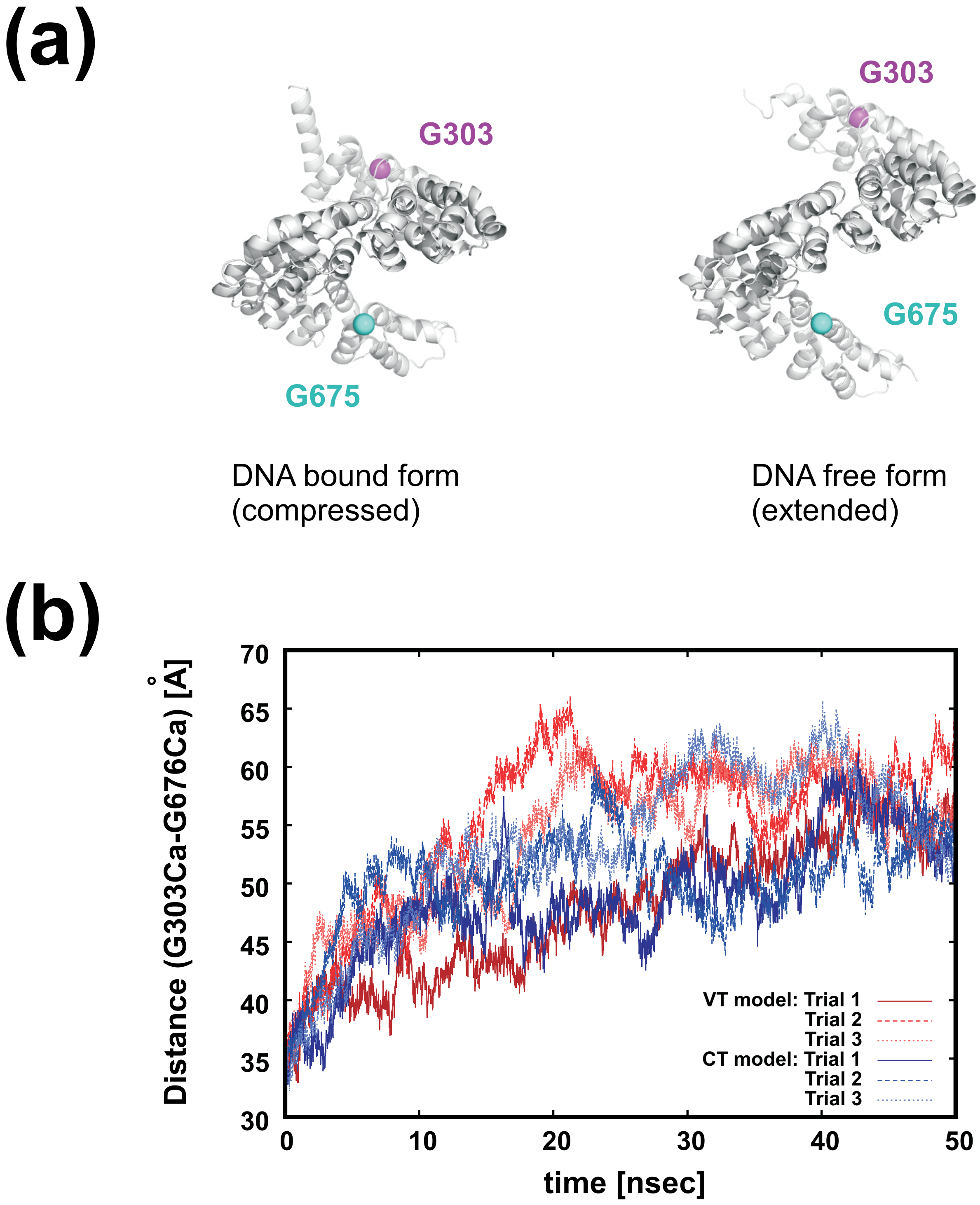

(a)
G303
G675
DNA bound form
(compressed)
G303
G675
DNA free form
(extended)
(b)
Distance (G303Ca-G676Ca) [Å]
time [nsec]
70
65
60
55
50
45
40
35
30
0
10
20
30
40
50
VT model: Trial 1
Trial 2
Trial 3
CT model: Trial 1
Trial 2
Trial 3

Fig. S4-1

(c)

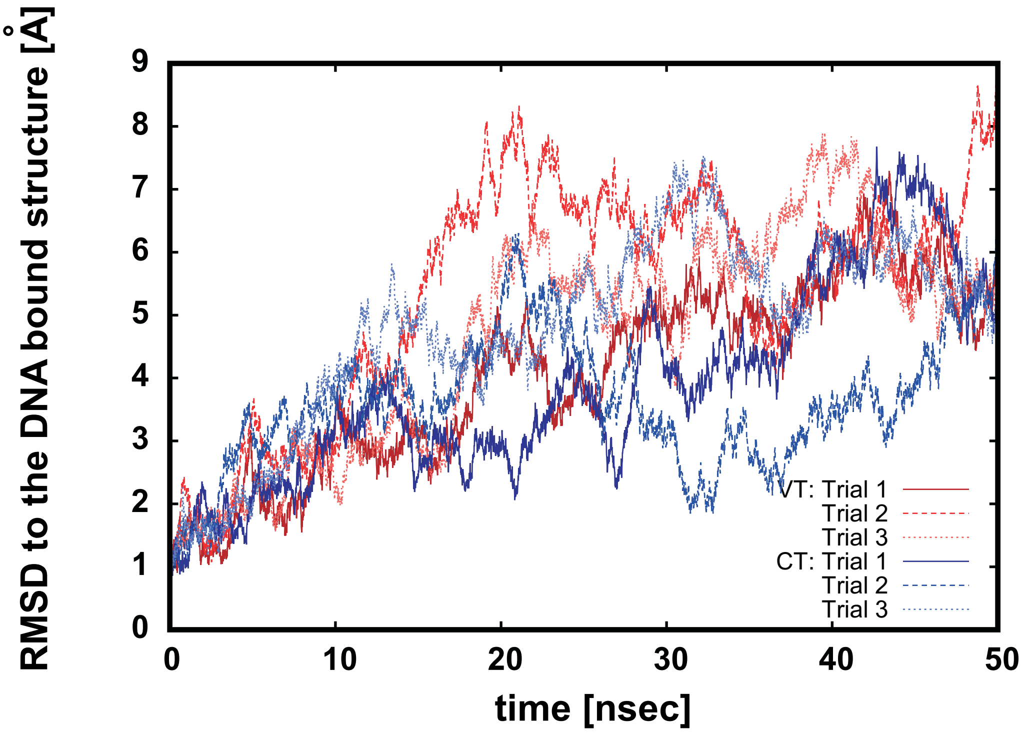
RMSD to the DNA bound structure [Å]
time [nsec]
0
1
2
3
4
5
6
7
8
9
0
10
20
30
40
50
VT: Trial 1
Trial 2
Trial 3
CT: Trial 1
Trial 2
Trial 3

(d)

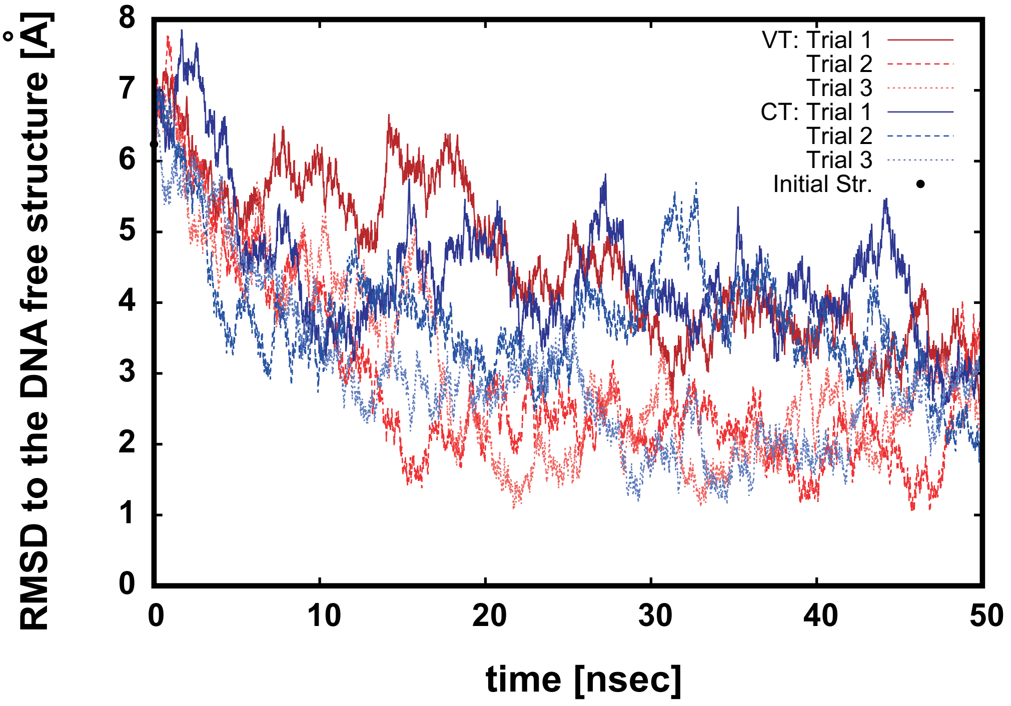
RMSD to the DNA free structure [Å]
time [nsec]
0
1
2
3
4
5
6
7
8
0
10
20
30
40
50
VT: Trial 1
Trial 2
Trial 3
CT: Trial 1
Trial 2
Trial 3
Initial Str.

Fig. S4-2

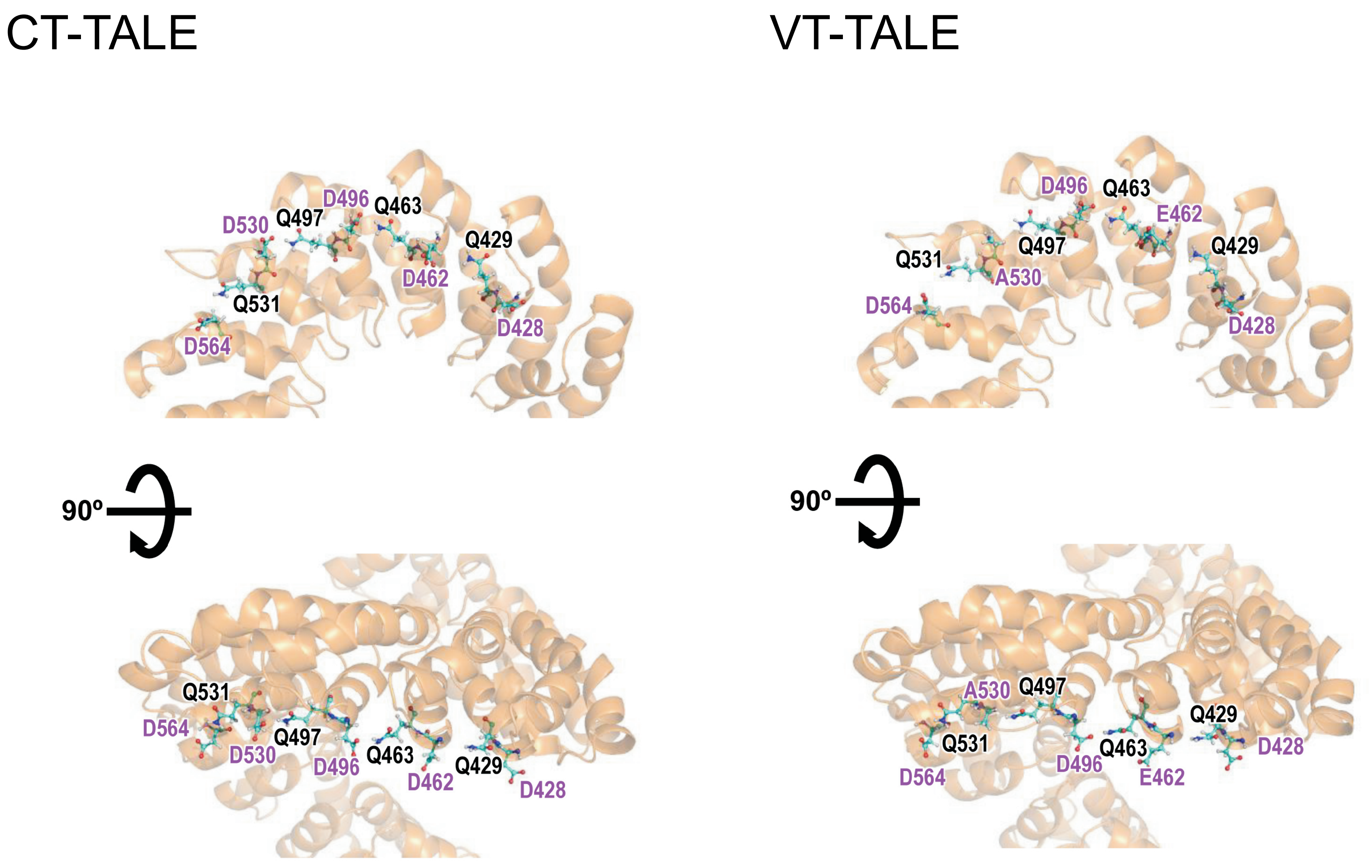
CT-TALE
VT-TALE
D530
Q497
D496
Q463
Q429
D462
Q531
D428
D564
90º
Q531
D564
D530
Q497
D496
Q463
D462
Q429
D428
D496
Q463
E462
Q429
Q497
Q531
A530
D564
D428
90º
A530
Q497
Q429
Q531
D428
Q463
D496
E462
D564

Fig. S5

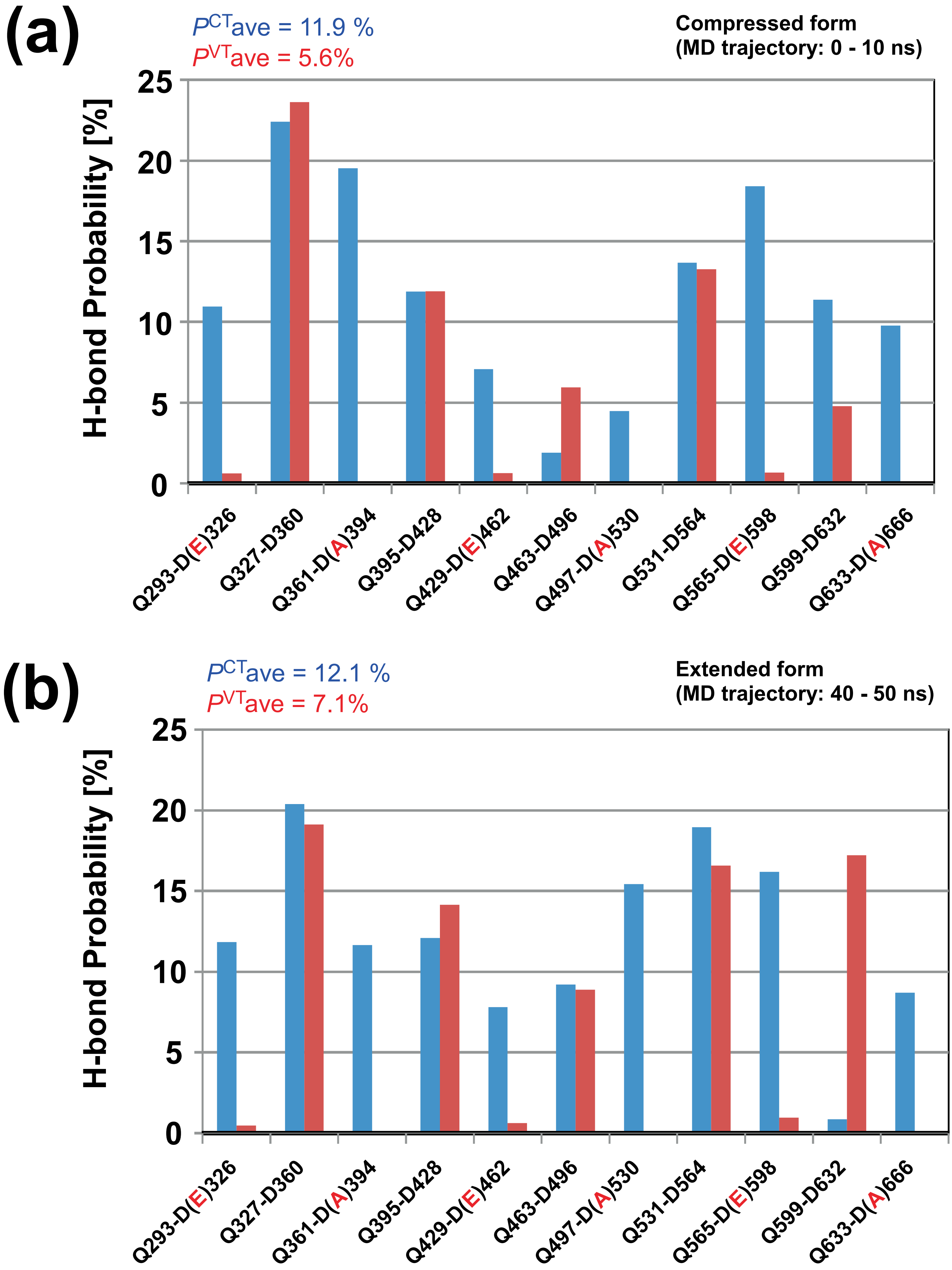
(a)
PCTave = 11.9 %
PVTave = 5.6%
Compressed form
(MD trajectory: 0 - 10 ns)
H-bond Probability [%]
25
20
15
10
5
0
Q293-D(E)326
Q327-D360
Q361-D(A)394
Q395-D428
Q429-D(E)462
Q463-D496
Q497-D(A)530
Q531-D564
Q565-D(E)598
Q599-D632
Q633-D(A)666
(b)
PCTave = 12.1 %
PVTave = 7.1%
Extended form
(MD trajectory: 40 - 50 ns)
H-bond Probability [%]
25
20
15
10
5
0
Q293-D(E)326
Q327-D360
Q361-D(A)394
Q395-D428
Q429-D(E)462
Q463-D496
Q497-D(A)530
Q531-D564
Q565-D(E)598
Q599-D632
Q633-D(A)666

Fig. S6
